# Novel mutant alleles of the starch synthesis gene *TaSSIVb*-*D* result in the reduction of starch granule number per chloroplast in wheat

**DOI:** 10.1186/s12864-017-3724-4

**Published:** 2017-05-08

**Authors:** Huijun Guo, Yunchuan Liu, Xiao Li, Zhihui Yan, Yongdun Xie, Hongchun Xiong, Linshu Zhao, Jiayu Gu, Shirong Zhao, Luxiang Liu

**Affiliations:** Institute of Crop Sciences, Chinese Academy of Agricultural Sciences/National Key Facility for Crop Gene Resources and Genetic Improvement/National Center of Space Mutagenesis for Crop Improvement, Beijing, 100081 China

**Keywords:** Wheat, TILLING, Mutant, *TaSSIVb*-*D*, Gene expression, Starch granule

## Abstract

**Background:**

Transient starch provides carbon and energy for plant growth, and its synthesis is regulated by the joint action of a series of enzymes. Starch synthesis IV (SSIV) is one of the important starch synthase isoforms, but its impact on wheat starch synthesis has not yet been reported due to the lack of mutant lines.

**Results:**

Using the TILLING approach, we identified 54 mutations in the wheat gene *TaSSIVb*-*D*, with a mutation density of 1/165 Kb. Among these, three missense mutations and one nonsense mutation were predicted to have severe impacts on protein function. In the mutants, *TaSSIVb*-*D* was significantly down-regulated without compensatory increases in the homoeologous genes *TaSSIVb*-*A* and *TaSSIVb*-*B*. Altered expression of *TaSSIVb*-*D* affected granule number per chloroplast; compared with wild type, the number of chloroplasts containing 0–2 granules was significantly increased, while the number containing 3–4 granules was decreased. Photosynthesis was affected accordingly; the maximum quantum yield and yield of PSII were significantly reduced in the nonsense mutant at the heading stage.

**Conclusions:**

These results indicate that *TaSSIVb*-*D* plays an important role in the formation of transient starch granules in wheat, which in turn impact the efficiency of photosynthesis. The mutagenized population created in this study allows the efficient identification of novel alleles of target genes and could be used as a resource for wheat functional genomics.

**Electronic supplementary material:**

The online version of this article (doi:10.1186/s12864-017-3724-4) contains supplementary material, which is available to authorized users.

## Background

Starch granules in plants are classified into two types according to their physiological role. Some starch granules are stored for long periods in reserve tissues as storage starch. This type of starch is an important source of carbohydrates for human beings. Other types of starch are stored temporarily as transient starch to provide carbon and energy for plant growth. Transient starch is synthesized in chloroplasts during the day and consumed during the night. Storage starch granules are synthesized in the plastid stroma by joint action of ADP-glucose pyrophosphorylases (AGPase), starch synthases (SS), starch branching enzymes (SBE), starch debranching enzymes (DBE) and their isoforms; however, the synthesis and regulation of transient granules hold a specific feature [1]. Some synthase isoforms that synthesize storage starch are different from those that synthesize transient starch.

AGPase is the first and rate-limiting enzyme in the biosynthesis of starch, it catalyzes the synthesis of ADP-glucose, which is the substrate of amylose starch, and over expression of the *AGPase* gene enhances the rate of starch biosynthesis [2–4]. Amylose, one of the main forms of starch, is synthesized from the substrate, ADP-glucose, by granule-bound starch synthase (GBSS). The isoform GBSS2 is responsible for amylose synthesis in leaves and other non-storage tissues [5–8], whereas amylopectin, the other major form of starch, is synthesized by the coordinated actions of AGPase, SS, and SBE. Five soluble SS isozymes, SSI, SSII, SSIII, SSIV, and SSV are found in plant genomes [9, 10]; and among them, SSI, SSII, and SSIII are involved in amylopectin elongation [11]. Isoform SSI is highly expressed in plant leaves [12, 13]. Two or three isoforms of SSII and SSIII are found in the genomes of different species, and at least one isoform is expressed in leaves and regulates starch granule biosynthesis [14–17]. Only one SSIV isoform is found in the *Arabidopsis* genome; *AtSSIV* is involved in the initiation of starch granule formation in leaves [18, 19]. Overexpression of *AtSSIV* increases the levels of starch accumulation by 30–40% and results in a higher rate of growth [20]; *SSIV* mutants have remarkably decreased numbers of granules and abnormal granules [18, 19, 21].

The functions of SSIV isozymes in the leaves of cereal crops are not as clear as in *Arabidopsis*. Two SSIV isoforms, SSIVa and SSIVb, are found in rice. *SSIVa* is mainly expressed in the endosperm and is responsible for starch accumulation in seeds, whereas *SSIVb* is mainly expressed in leaves at early development stages and is responsible for leaf granule biosynthesis [12, 14, 22]. It also has overlapping and crucial roles with SSIIIa in rice seeds in determining granule morphology and in maintaining the amyloplast envelope structure [23]. *ZmSSIV* is highly expressed in the embryo, endosperm and pericarp in maize. In wheat, only one isoform of SSIV has been identified until now. This gene is located on the long chromosome arm of homologous group I and shows high similarity with *OsSSIVb*, so this isoform is named *TaSSIVb* [12, 24]. *TaSSIVb* is preferentially expressed in leaves and is not regulated by the circadian clock [24]. *TaSSIVb* is also highly expressed at the middle stages of seed development [25, 26]; however, the impact of *TaSSIVb* on wheat leaf and seed granule characteristics at different growth stages have not yet been reported due to the lack of mutant lines.

TILLING (Targeting-Induced Local Lesions IN Genomes) is a powerful approach for novel allele identification and has been used to screen mutagenized plant populations for novel alleles of target genes [27–34]. Hundreds of missense and nonsense mutant alleles of key starch synthesis-related wheat genes, such as *TaSSII*, *TaSBEIIa*, *TaSBEIIb*, have been identified using this platform [35, 36]. A hexaploid wheat line containing two *waxy* homozygous mutations created through TILLING and a pre-existing deletion of a third *waxy* homoeolog displays a near-null phenotype [37]. Novel alleles of *TaSBEII* have been identified in both durum wheat and bread wheat. Double mutant lines combining a *TaSBEIIa*-*A* mutation with a *TaSBEIIa*-*B* mutation in durum wheat and triple mutant lines combining mutations in *TaSBEIIa*-*A*, *TaSBEIIa*-*B* with *TaSBEIIa*-*D* have a significant increase in both amylose and resistant starch content [38–42]. Using this approach to identify novel alleles of *TaSSIVb* would be helpful in characterizing its role in transient starch granule synthesis in wheat leaves.

In this paper, we used the TILLING approach to identify novel alleles of *TaSSIVb*–*D* with mutations in functional regions in an Ethyl methanesulphonate (EMS) mutagenized population derived from an elite hexaploid wheat cultivar. Homozygous mutants were used to investigate the specific role of *TaSSIVb*-*D* in starch granule synthesis in leaves at different stages of growth. We found that *TaSSIVb*-*D* mutations decrease gene expression and the number of starch granules in leaves.

## Results

### Novel alleles of *TaSSIVb*-*D* in a mutagenized population

**Fig. 1:**
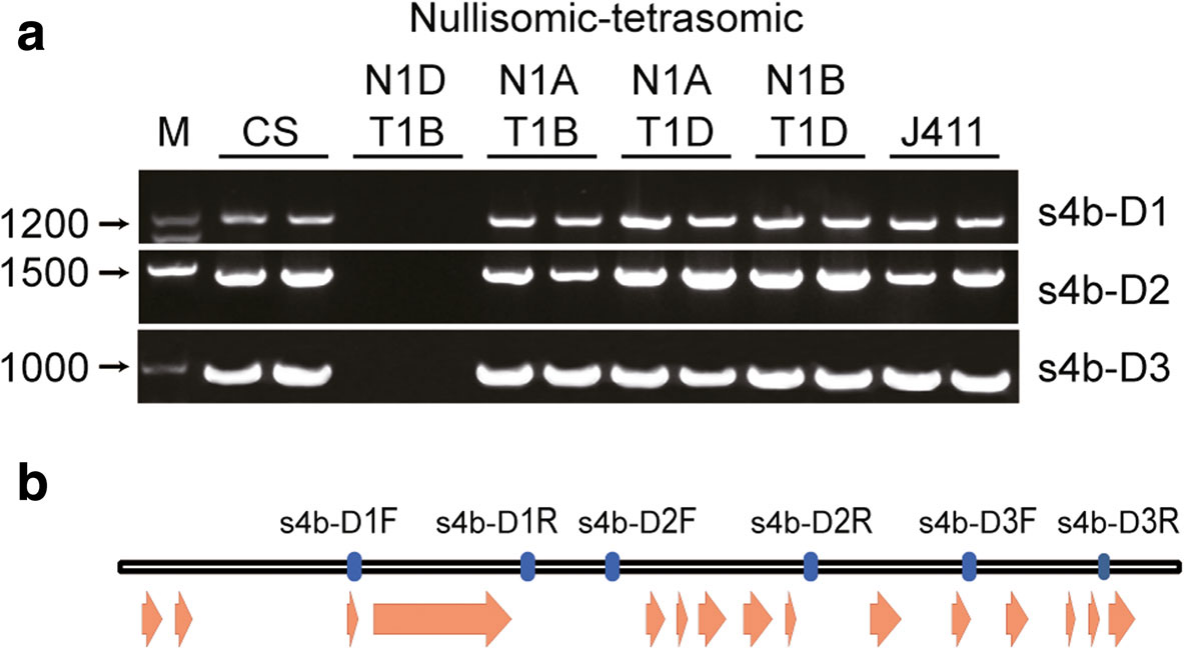
Validation of subgenome-specific *TaSSIVb*-*D* primers using the Chinese Spring nullisomic-tetrasomic lines. **a** Products from the Chinese Spring (CS), nullisomic-tetrasomic lines (N1AT1B, N1AT1D, N1BT1D and N1DT1B) and wild type (J411) amplified using the three different sets of *TaSSIVb*-*D*-specific primer pairs shown in b. *TaSSIVb*-*D* is absent from N1DT1B. **b** Diagram of D subgenome primers. The black rectangle represents the gene sequence; orange arrowheads indicate exon regions; blue rectangles represent the location of each primer

Three primer sets corresponding to *TaSSIVb*-*D* were designed for allele mining (Table 1), and their specificities were validated using nullisomic-tetrasomic lines (Fig. 1a). The location of these primers on the *TaSSIVb*-*D* sequence is shown in Fig. 1b.

**Table 1:** Primer sequences used for allele mining

Name	Forward (5’-3’)	Reverse (5’-3’)
s4b-D1	ACTAAAACCCACTTTGCGAC	GGTAGGAATGATACAGAACACC
s4b-D2	CTGCAAAAAATTGTCTAAAAGCTAC	CATGCTTTGAAATTATCTACTTTCG
s4b-D3	ACCAGAAATTCAGGTGCGTT	TGAGTCGTGTTGTGCCCG

In the mutagenised population, 54 mutations were identified with a mutation density of 1/165 Kb. Among these mutations, 52 are G to A (61.12%) or C to T (35.19%) transition point mutations, and the other 2 are T insertions. Of these mutations, 26 are in the coding region (Table 2), including 1 nonsense mutation (E054-13), 15 missense mutations and 10 silent mutations. The remaining 26 are located in introns and include 2 splice junction mutations.

**Table 2:** Mutations identified in the coding region of the Ta*SSIV b*-*D* gene

Line	Allele	Mutation Type	Effect	PSSM	SIFT
E334	G1783A	Silent			
E1239	G1813A	Missense	E181K	4.4	0.6
E469	G1822A	Missense	E183K	−6	0.42
E939	G1848A	Silent			
E1337	G1863A	Silent			
E2-2-226	T1880C	Missense	L203S	26.7	0
E2-2-437	T1880C	Missense	L203S	26.7	0
E468	G1890A	Silent			
E1245	G1920A	Silent			
E391	G1975A	Missense	D235N		
E137	G2008A	Missense	D246N	0.4	0.96
E054-13	C2077T	Nonsense	Q269-Stop		
E2-2-193	C2143T	Missense	L291F	0.6	0.02
E2-2-95	C2152T	Missense	L294F		
E570	A2219G	Missense	Q316R	8.4	0
E1347	C2353T	Silent			
E783	G2402A	Missense	S377N		
E236	G2413A	Silent			
E996	C3516T	Silent			
E2-2-181	G3536A	Missense	V469I		
E054-9	C3740T	Intronic			
E1226	G3932A	Missense	V550I	0.9	0.09
E972	C3950T	Missense	H556Y	27.5	0
E930	G5872A	Silent			
E1373	C5878T	Silent			
E1137	G6327A	Missense	S833N	12	0

Nucleotide changes are numbered relative to the start codon ATG

Using SIFT program, five missense mutations (E2-2-226 and E2-2-437, E2-2-193, E570, E972, E1137) are predicted to have a severe impact on protein function. Based on the PSSM program, three missense mutations (E2-2-226 and E2-2-437, E972, E1137) are predicted to have a severe impact. As the nonsense mutant E054-13 results in the loss of both the starch catalytic domain and glycosyltranferase domain, it might have a severe impact on protein function. Because the E1137 mutation is located in the glycosyltranferase domain which has less similarity with other SS isoforms [24], and might have distinct functions from other starch synthases, mutants E1137 and E054-13 were selected for analysis of gene expression and starch granule number.

### The expression of *TaSSIVb* in mutant leaves at different growth stages

**Fig. 2:**
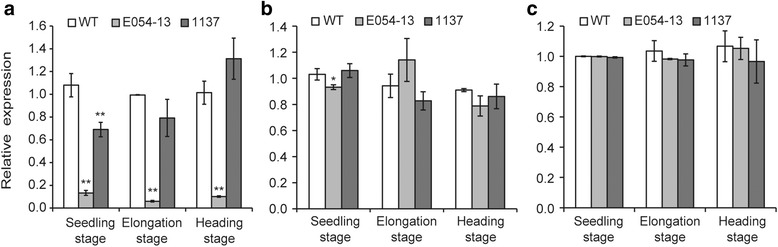
Expression patterns of the three homeologous *TaSSIVb* genes in leaves at different stages. **a** Relative expression level of *TaSSIVb*-*D*; **b** Relative expression level of *TaSSIVb*-*A*; **c** Relative expression level of *TaSSIVb*-*B*. Error bars represent standard deviation; WT: wild type; * means significantly different from WT at the *P* = 0.05 level and ** means significantly different from WT at the *P* = 0.01 level based on Student’s *t* test

Specific primer sets for each sub-genome (Additional file 1: Table S1, Additional file 2: Figure S1) were used to analyze the pattern of *TaSSIVb* gene expression. In wild type, gene expression profiling revealed non-significant differences in expression between different growth stages (Fig. 2). In the mutants, *TaSSIVb*-*D* was down-regulated, and *TaSSIVb*-*A* and *TaSSIVb*-*B* did not show significant compensatory responses. The expression of *TaSSIVb*-*D* in the nonsense mutant E054-13 was reduced by ~8-fold compared with the wild type at all three growth stages, whereas in E1137, its expression level gradually increased with plant growth and only showed a significant reduction (36.12%) at the seedling stage (Fig. 2a). In contrast, no significant differences in the expression of *TaSSIVb*-*A* (Fig. 2b) and *TaSSIVb*-*B* (Fig. 2c), were detected in the two *TaSSIVb*-*D* mutants at all three stages, except for *TaSSIVb*-*A* expression in the E054-13 mutant at the seedling stage (9.55% reduction).

### Variation in the number of starch granules in the chloroplast

**Fig. 3:**
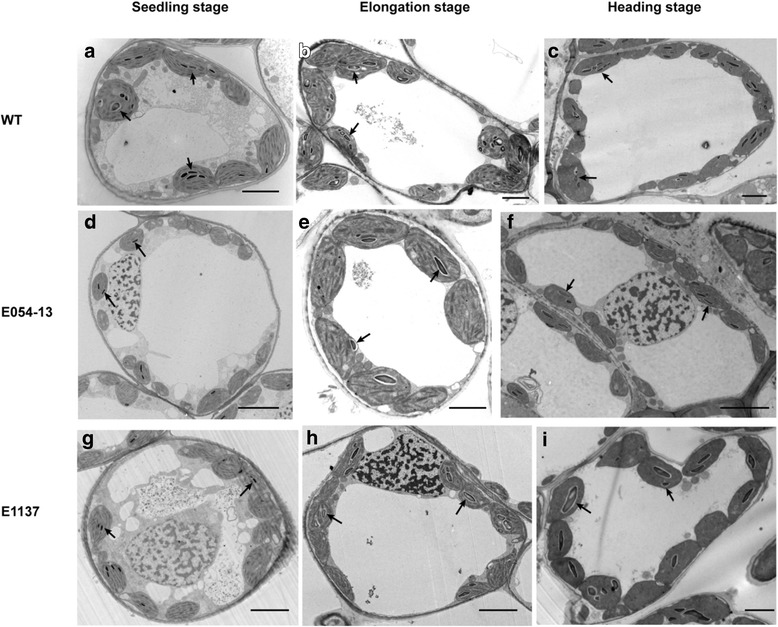
Starch granules observed in the chloroplasts of wild type (**a**, **b**, **c**) and *TaSSIVb*-*D* E054-13 (**d**, **e**, **f**) and E1137 (**g**, **h**, **i**) mutants by transmission electron microscopy. Arrows indicate starch granules, bars = 5 μm

We investigated starch granule characteristics in the chloroplasts of wild type and the two mutants at three growth stages (Fig. 3). Granule number varied plast by plast in each genotype, with numbers ranging from 0 to 8 and more than 90% of chloroplasts contained no more than 4 granules (Additional file 1: Table S2). The difference between the wild type and mutants was mainly in the number of chloroplasts containing no more than 4 granules.

**Fig. 4:**
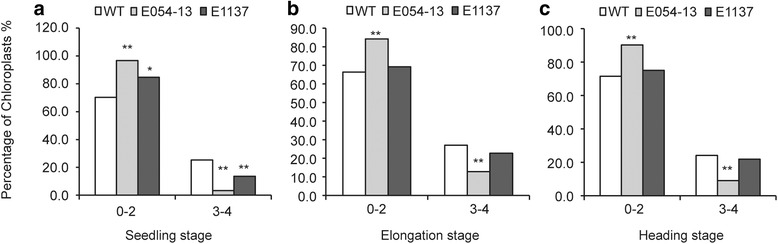
Percentage of chloroplasts in wild type (WT) and *TaSSIVb*-*D* mutants (E054-13 and E1137) containing 0–2 and 3–4 granules at three different growth stages. **a** Seedling stage; **b** Elongation stage; **c** Heading stage. The percentage of chloroplasts equals the number chloroplasts containing 0–2 and 3–4 starch granules divided by the total number of chloroplasts observed. * means significantly different from WT at the *P* = 0.05 level and ** means significantly different from WT at the *P* = 0.01 level

The biggest difference in granule number per chloroplast was observed at the seedling stage, and mutation of *TaSSIVb*-*D* led to a reduction in granule number. The percentage of chloroplasts with 0–2 granules in both mutants, E054-13 (96.70%) and E1137 (84.70%), were significantly higher compared with wild type (70.30%) (Fig. 4), whereas at the elongation and heading stages, the difference between E1137 and wild type became smaller; only the E054-13 mutant had a significantly higher percentage of chloroplasts with 0–2 and 3–4 granules (17.74% and 18.72% higher than wild type, respectively). In contrast, the percentage of chloroplasts containing 3–4 granules was lower in the mutants than in wild type. At the seedling stage, the percentage of chlorplasts in E054-13 and E1137 with 3–4 granules was 22.00% and 10.3% lower than wild type, respectively. At the elongation and heading stages, only the E054-13 mutant was significantly different from wild type; the percentage of chlorplasts with 3–4 granules was 14.26% and 15.05% lower than wild type, respectively. The observation of the chloroplast granule number phenotype at all stages for the E054-13 mutant and only at the seedling stage for the E1137 mutant is consistent with the lower levels of *TaSSIVb*-*D* gene expression in the mutants at these stages (Fig. 2).

### Photosynthesis parameters and the efficiency of PSII

**Fig. 5:**
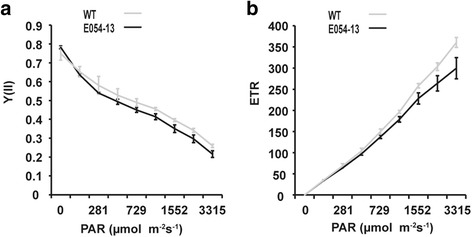
Y(II) (**a**) and ETR (**b**) fast light curves for wild type and the *TaSSIVb*-*D* E054-13 nonsense mutant at the heading stage. Error bars represent standard deviation

As transient starch in chloroplasts provides carbon and energy for plant growth, we deduced that photosynthesis might be affected by reduced granule numbers. Granule numbers in the mutant E054-13 were more severely reduced than those in E1137, and the impact on photosynthesis parameters are expected to be more severe. Therefore, photosynthesis parameters in the mutant E054-13 and the wild type at the heading stage were compared to determine the impact of reduced granule number. We found that the maximum quantum yield (*F*_*v*_/*F*_*m*_) and yield of PSII (Y(II)) of E054-13 were significantly lower than in wild type (Table 3), and differences between the mutant and wild type in both Y(II) and electron transport rate (ETR) increased with increasing photosynthetically active radiation (PAR) (Fig. 5). As Y(II) provides the effective quantum yield and could be used to estimate the effective portion of absorbed quanta; while ETR provides information for plant stress reaction, so the results indicates that mutation of *TaSSIVb*-*D* leads to a decrease in relative quantum yield and has a negative effect on PSII efficiency.

**Table 3:** Changes in chlorophyll fluorescence parameters in the *TaSSIVb*-*D* E054-13 nonsense mutant at the heading stage

Genotype	Y(II)	*F*_*v*_/*F*_*m*_
Wild type	0.641 ± 0.0019	0.818 ± 0.0175
E054-13	0.617 ± 0.0020 **	0.622 ± 0.0274 **

Y(II) and *F*_*v*_/*F*_*m*_ were measured in dark-adapted plants, and Photosynthetically Active Radiation (PAR) equals 281. Values are means ± standard deviation

** significantly different from the wild type at the *P* < 0.01 level based on Student’s *t* test

## Discussion

### Conserved and functional regions of *TaSSIVb*

As one of the isoforms of the soluble starch synthesis family, wheat SSIV contains the starch catalytic domain (GT-5) and glycosyltranferase domain (GT-1). GT-5 (amino acids 422–661) is more conserved than GT-1 (amino acids 706–886) between SS isoforms [24]. Two mutations that we identified in this study, E972 and E1137, are located in GT-5 and GT-1, respectively, and in the wild type these amino acids are involved in the formation of β-sheets (data not shown). In contrast the N-terminus (amino acids 1–405) is distinct from other SS isoforms, and this region contains two coiled-coil domains and a 14-3-3-protein binding site [24]. The missense mutation E2-2-226 and the truncation mutation E054-13 are located in this region. These novel alleles could be very useful in understanding the specific functions of SSIV in starch granule synthesis in wheat. The gene expression patterns and alteration of granule number in the E1137 and E054-13 mutants demonstrate the important roles of these predicted functional regions. The E054-13 truncation mutation has a more severe effect than the missense mutation; *TaSSIVb* gene expression is reduced by 5 ~ 13-fold compared with E1137 (Fig. 2), and granule number is also significantly decreased compared with E1137 (Additional file 1: Table S2).

### Expression of *TaSSIVb* homoeologs

Wheat has three sub-genomes, and the literature shows that the contribution of each homeologous gene can be different. For example, of the three *SSIIa* genes, *SSIIa* on the B genome has the largest contribution to amylopectin structure than those on the other two genomes [43]. Each *waxy* gene encoding GBSSI has different effects on amylopecitin characteristics [37, 44, 45]. SS isoform SSIV is encoded by three homeologous genes located on group I chromosomes [24] and the effect of each gene and their interactions is not clear. Here we designed homeologous-specific primers to determine the gene expression patterns of the three *SSIV* genes. In *TaSSIVb*-*D* mutants, decreased expression of *TaSSIVb*-*D* did not result in significant changes in the expression of *TaSSIVb*-*A* or *TaSSIVb*-*B*. This is similar to what was observed in the single null mutant of *SBEIIa*; a mutation in one sub-genome does not result in appreciably different expression in the other two sub-genomes [46]. However, the identification of mutations in *TaSSIVb*-*A* and *TaSSIVb*-*B* is needed to analyze the interaction between homoeologous copies or their dosage effects.

### Expression of *TaSSIV*-*D* in wheat leaves

The function of *AtSSIV*, which it is highly expressed in leaves in *Arabidopsis* is very well understood. In wheat, the expression of *TaSSIVb* is tissue-dependent, and is much more highly expressed in leaves than in the endosperm [24]. Our results are consistent with these reports; all three homeologous genes are highly expressed in leaves during the entire growth period. There is also evidence that *TaSSIVb* is expressed during seed development [24–26]. In ongoing research we are investigating the accumulation of storage starch in both the E054-13 and E1137 mutants to elucidate the role of *TaSSIVb* in storage starch synthesis.

### Starch granule synthesis

Starch granules are initiated early in the development of young leaves in *Arabidopsis*. Because more starch granules are observed in immature leaves than in mature leaves [47], it is believed that SSIV functions in granule initiation at earlier growth periods [21]. The *ssIV* mutant in *Arabidopsis* has reduced granules in most chloroplasts [18, 21]. The results of this study demonstrate that in young wheat leaves *TaSSIV*-*D* has a function similar to *AtSSIV*, and it is involved in initiation of transitory starch synthesis. At the seedling stage, the expression patterns of both the E054-13 and E1137 *TaSSIV*-*D* mutants are down regulated. In the E1137 mutant, the significant reduction of granule number is only observed when the expression of *TaSSIV*-*D* is reduced; while in the nonsense mutant, starch granules are reduced at all the three stages. This indicates that reduction of starch granule number results from the mutation of *TaSSIV*-*D* and not other genes.

### Effect of homeologous genes

In many cases, homeologous genes from different sub-genomes of wheat have dosage effects on phenotypes. Only double or triple mutants of *SSIIa* have severe effects on starch properties [48, 49], and single-gene mutants do not show significant phenotypes [38]. However in single *TaSSIV*-*D* gene mutants, gene expression level, granule number, relative quantum yield and PSII efficiency are significantly reduced with respect to the wild type. It has been reported that single mutants of the genes *SBEIIa* and *gpc* (grain protein content) also show significant phenotypic differences compared with the wild type [46, 50], so not only double and triple mutants, but also single mutants can lead to phenotypic changes in hexaploid wheat.

### Relationship between transient starch content and photosynthesis

It has been reported that the capacity for starch synthesis and the rate of photosynthesis are positively correlated [51]. A starchless tobacco mutant shows about 50% inhibition of maximum photosynthetic capacity [52]. Mutation of *SSIV* in *Arabidopsis* also has negative effects on photosynthesis efficiency [53]. Our *SSIV* mutant in wheat shows the same effects; photosynthesis efficiency is significantly reduced. This is likely because *SSIV* mutants accumulate high concentrations of the substrate ADPglucose [21], which leads to photooxidative stress and decreased photosynthesis efficiency [53].

## Conclusions

We identified novel alleles, including missense and nonsense mutations, of the starch synthesis gene, *TaSSIVb*-*D*. Gene expression, granule number and photosynthetic parameters are down-regulated or reduced in the missense mutant E1137 and the nonsense mutant E054-13, and the impact of E054-13 is much more severe than that of E1137. This demonstrates *TaSSIVb*-*D* plays an important role in the formation of transient starch granules in wheat, and mutation of *TaSSIVb*-*D* results in remarkable reduction in transitory starch granules in leavies. The mutagenized population generated in this study could be used as a resource for gene functional research.

## Methods

### Materials

Wheat (*Triticum aestivum* L.) cultivar Jing411 was used as the wild type (WT) to create the mutagenized population. Homozygous mutant lines grown in the field with normal management were used for RT-qPCR and phenotyping.

### Development of the wheat TILLING population

Seeds from the wild type Jing411 were soaked in tap water for 10 h at 20°C, then incubated in 1.5% EMS in 0.1 M sodium phosphate buffer (pH7.0) for 4 h or 6 h at 20°C. This was followed by washing with running tap water for 4 h at room temperature. The mutagenized seeds (M_1_ generation) were planted directly in the field and self-fertilized. The M_2_ was developed using the single-seed descent (SSD) method.

The mutagenized population consists of 3058 M_2_ plants, including 1441 individuals from the 4 h 1.5% EMS treatment and 1617 from the 6 h treatment. A young leaf from each individual was sampled at the seedling stage for genomic DNA extraction using the DNA-quick Plant System kit (Tiangen Biotech, Beijing, China). DNA concentration was measured with the NanoDrop2000 spectrophotometer (Thermo Fisher Scientific, USA) according to the manufacturer’s instructions, and the final concentration was adjusted to 50 ng/μl. Individual DNA samples were pooled 2-fold into 96-well plates for TILLING screening, which was done according to Till *et al*. [54].

### Primer design

The sequence of *TaSSIVb* (DQ400416) was downloaded fromhttps://www.ncbi.nlm.nih.gov/nuccore/DQ400416, and the sequences of *TaSSIVb*-*A*, *TaSSIVb*-*B* and *TaSSIVb*-*D* were obtained through alignment with long arm of chromosome 1A, 1B and 1D (https://wheat-urgi.versailles.inra.fr/Seq-Repository). *TaSSIVb*-*D* was used as the target gene for mining and phenotyping novel alleles. Primer Premier 5.0 (Premier Biosoft International, Palo Alto, CA) was used to design homoeolog-specific TILLING and qPCR primers (Table 1 and Additional file 1: Table S1), and their specificities were validated using Chinese Spring nullisomic-tetrasomic lines (N1AT1B, N1AT1D, N1BT1D and N1DT1B; Fig. 1a and Additional file 2: Figure S1).

### Mutation screening

Each primer used for TILLING detection was synthesized with and without fluorescent dye label. Unlabeled primers (Sangon Biotech, China), forward primers labeled with the fluorescent dye IRD700 and reverse primers labeled with the fluorescent dye IRD800 (Bioneer, China) were mixed with the ratio of unlabeled forward: labeled forward: unlabeled reverse: labeled reverse = 4: 1: 1: 4. PCR amplification and polyacrylamide gel electrophoresis were carried out as previously described [54]. Identified mutations were sequenced to verify nucleotide variation. Mutation density was calculated by dividing the total number of mutations by the total length of the sampled DNA sequence (length of the amplified fragment × number of individuals sampled). Totally 3058 individual plants were screened for each primer set, and the amplicon size of primer set s4b-D1, s4b-D1 and s4b-D1 was 1191, 1361 and 960 bp respectively. As DNA products at the top and bottom of the gel were difficult to detect, we subtracted 100 bp from the 5’ terminus and 100 bp from the 3’ terminus of each fragment.

The PARSESNP (Project Aligned Related Sequences and Evaluate SNPs; http://blocks.fhcrc.org/proweb/parsesnp/) and SIFT (Sorting Intolerant from Tolerant; http://sift.bii.a-star.edu.sg/) programs were used to predict the severity of each mutation. Mutations with PSSM >10 or SIFT <0.05 are predicted to have a severe effect on protein function [55, 56].

### RT-qPCR

The effects of mutations on the expression of *TaSSIVb* gene homoeologs was evaluated for leaves at the seedling, elongation and heading stages. Total RNA was extracted from three individual plant leaves, and cDNA was synthesized using the FastQuant RT kit (With gDNase, Tiangen Biotech) according to the manufacturer’s instructions. RNA extraction and cDNA synthesis was done for three biological replicates per genotype with three technical replicates for each biological replicates, and their concentrations measured with the NanoDrop2000 spectrophotometer, the same volume and concentration of normalized template was used for the next step experiment. RT-qPCR was performed on a CFX96 system using the SsoFast EvaGreen Supermix kit (Bio-Rad), PCR was performed at 94°C for 3 min, followed by 40 cycles at 94°C for 30 s, annealing for 30 s, and 72°C for 10 s, and then a melt curve stage. Concentration of each primer was 0.3 μmol, amplification efficiency of the three genes ranged from 97% to 100%. The actin gene used as a control was the same as described by Gu *et al*. [57]. The relative expression level was calculated with ΔΔ*C*t method according to the CFX96 manual, data were analyzed by one-way ANOVA using Microsoft Excel software.

### Electron microscopy

At the seedling, elongation, and heading stages, leaves of mutants and WT at the same developmental stage were sampled with 3 replicates for transmission electron microscope analysis. Each sample was processed as described in Guo *et al*. [58] and photographed with a transmission electron microscope (HT-7700, Hitachi, Japan). Starch granule numbers per chloroplast were calculated, and data were analyzed using the SPSS software [59].

### Chlorophyll fluorescence parameters

A pulse amplitude modulation fluorometer (MINI-PAM, Heinz Walz, Effeltrich, Germany) was used to measure chlorophyll fluorescence parameters. At the seedling, elongation, and heading stages, individual plants at the same developmental stage were selected for measurements with three biological and three technical replicates. From 10:00-11:00 am on the same day, after dark adaption by Dark Leaf Clip for 30 min, the value of *F*_*v*_/*F*_*m*_ and Y(II) were measured with a photosynthetically active radiation (PAR) of 281 μmol m^-2^s^-1^ according to the manufacturer’s instructions. The response of photosynthetic fluorescence parameters to light intensity changes was determined using rapid light curves. Imaging Win software (Heinz Walz, Effeltrich, Germany) was used to collect and analyze fluorescence parameter data. Data were analyzed by one-way ANOVA with Microsoft Excel software.

## Additional files

Additional file 1: Table S1. Primers used for RT-qPCR. **Table S2.** Percentage of chloroplasts containing different numbers of starch granules. (DOCX 19 kb)

Additional file 2: Figure S1. Validation of sub-genome-specific RT-qPCR primers using Chinese Spring nullisomic-tetrasomic lines. CS: Chinese Spring; N1DT1B, N1AT1B, N1AT1D, and N1BT1D: Chinese Spring nullisomic-tetrasomic lines; J411: wild type; s4b-qD: *TaSSIVb*-*D*-specific primers; s4b-qA: *TaSSIVb*-*A*-specific primers; s4b-qB: *TaSSIVb*-*B*-specific primers. (DOCX 1759 kb)

## Abbreviations

AGPase: ADP-glucose pyrophosphorylases
DBE: Starch debranching enzymes
EMS: Ethyl methanesulphonate
ETR: Electron transport rate
GBSS: Granule-bound starch synthase
*gpc*: Grain protein content
GT-1: Glycosyltranferase domain
GT-5: Starch catalytic domain
PAR: Photosynthetically active radiation
PARSESNP: Project aligned related sequences and evaluate SNPs
RT-qPCR: Reverse transcription quantitative PCR
SBE: Starch branching enzymes
SIFT: Sorting intolerant from tolerant
SPSS: Statistical package for the social sciences
SS: Starch synthases
SSD: Single-seed descent
SSIV: Starch synthesis IV
TILLING: Targeting induced local lesions in genomes
WT: Wild type
Y(II): Yield of PSII
