# Supplementary material for: Novel mutant alleles of the starch synthesis gene TaSSIVb-D result in the reduction of starch granule number per chloroplast in wheat
Source: BMC Genomics. 2017 May 8;18:358. doi: 10.1186/s12864-017-3724-4 (PMC5422989; doi:10.1186/s12864-017-3724-4)
Supplement: Supplementary file 1 — Primers used for RT-qPCR. Table S2. Percentage of chloroplasts containing different numbers of starch granules. (DOCX 19 kb) [file 12864_2017_3724_MOESM1_ESM.docx]

**Additional file 2**

**Table S1 Primers used for RT-qPCR**

| Name | Forward (5'-3') | Reverse (5'-3') |
| --- | --- | --- |
| s4b-qA | TTCCAAGCATGTAATTGT | TTTATGAACCATTTCTCGC |
| s4b-qB | GGTTGTCAGAAATAGATTC | CCTTTAGAACATCAAACTCT |
| s4b-qD | CCGCTTCCAAGCATGTAACAGC | CCAGTAACCATCCATCAATTAG |

**Table S2 Percentage of chloroplasts containing different numbers of starch granules**

| **Stage** | **Genotype** | **Chloroplasts observed** | **Percentage of chloroplasts (%)^1^** | | | | | | | | |
| --- | --- | --- | --- | --- | --- | --- | --- | --- | --- | --- | --- |
|  |  |  | 0 | 1 | 2 | 3 | 4 | 5 | 6 | 7 | 8 |
| Seedling stage | WT | 411 | 29.70 | 20.40 | 20.20 | 14.60 | 10.7 | 1.70 | 0.70 | 1.70 | 0.20 |
|  | E054-13 | 1284 | 51.00 | 29.30 | 16.40 | 2.80 | 0.50 | 0.10 | 0 | 0 | 0 |
|  | E1137 | 881 | 34.50 | 29.30 | 20.90 | 10.30 | 3.30 | 1.40 | 0.20 | 0.10 | 0 |
| Elongation stage | WT | 921 | 22.91 | 25.62 | 17.92 | 14.77 | 12.27 | 4.45 | 1.19 | 0.76 | 0.11 |
|  | E054-13 | 829 | 38.72 | 29.31 | 16.16 | 8.20 | 4.58 | 2.29 | 0.60 | 0.12 | 0 |
|  | E1137 | 933 | 27.55 | 21.44 | 20.26 | 13.93 | 8.79 | 4.93 | 1.82 | 1.07 | 0.11 |
| Heading stage | WT | 950 | 20.32 | 25.05 | 26.21 | 16.42 | 7.68 | 3.16 | 0.84 | 0.32 | 0 |
|  | E054-13 | 1093 | 31.93 | 35.86 | 22.51 | 7.59 | 1.46 | 0.37 | 0.27 | 0 | 0 |
|  | E1137 | 806 | 25.68 | 23.33 | 26.05 | 14.27 | 7.57 | 1.86 | 0.99 | 0.25 | 0 |

^1^ The percentage of chloroplasts equals the number chloroplasts containing the indicated number of starch granules divided by the total number of chloroplasts observed.
