# Supplementary material for: Novel mutant alleles of the starch synthesis gene TaSSIVb-D result in the reduction of starch granule number per chloroplast in wheat
Source: BMC Genomics. 2017 May 8;18:358. doi: 10.1186/s12864-017-3724-4 (PMC5422989; doi:10.1186/s12864-017-3724-4)

**Figure S1** Validation of sub-genome-specific RT-qPCR primers using Chinese Spring nullisomic-tetrasomic lines. CS: Chinese Spring; N1DT1B, N1AT1B, N1AT1D, and N1BT1D: Chinese Spring nullisomic-tetrasomic lines; J411: wild type; s4b-qD: *TaSSIVb-D*-specific primers; s4b-qA: *TaSSIVb-A*-specific primers; s4b-qB: *TaSSIVb-B*-specific primers.


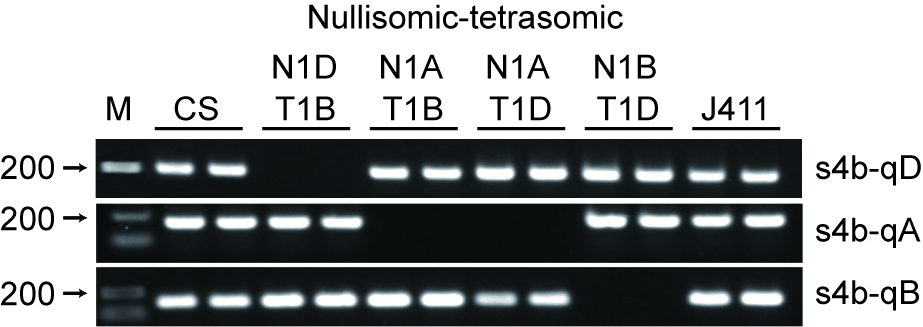

Supplement: Supplementary file 2 — Validation of sub-genome-specific RT-qPCR primers using Chinese Spring nullisomic-tetrasomic lines. CS: Chinese Spring; N1DT1B, N1AT1B, N1AT1D, and N1BT1D: Chinese Spring nullisomic-tetrasomic lines; J411: wild type; s4b-qD: TaSSIVb-D-specific primers; s4b-qA: TaSSIVb-A-specific primers; s4b-qB: TaSSIVb-B-specific primers. (DOCX 1759 kb) [file 12864_2017_3724_MOESM2_ESM.docx]
